# Efficacy and Safety of Intensity‐Modulated Radiotherapy Combined With Regorafenib With or Without Immune Checkpoint Inhibitors as Second‐Line Treatment for Advanced Hepatocellular Carcinoma: A Real‐World Cohort Study From a Single Center

**DOI:** 10.1002/cam4.71745

**Published:** 2026-04-10

**Authors:** Lingxia Xin, Zhiyu Li, Yirui Zhai, Liming Wang, Feng Ye, Yongkun Sun, Wen Zhang, Tiago Biachi de Castria, Sanjaya K. Satapathy, Vishal G. Shelat, Shaffer R. S. Mok, Jennifer Wu, Shulian Wang, Yueping Liu, Yongwen Song, Yuan Tang, Hao Jing, Hui Fang, Shunan Qi, Ningning Lu, Ye‐Xiong Li, Xinyu Bi, Bo Chen

**Affiliations:** ^1^ Department of Radiation Oncology, National Cancer Center/National Clinical Research Center for Cancer/Cancer Hospital, Chinese Academy of Medical Sciences (CAMS) and Peking Union Medical College (PUMC) Beijing China; ^2^ Department of Hepatobiliary Surgery, National Cancer Center/National Clinical Research Center for Cancer/Cancer Hospital, Chinese Academy of Medical Sciences (CAMS) and Peking Union Medical College (PUMC) Beijing China; ^3^ Department of Radiology, National Cancer Center/National Clinical Research Center for Cancer/Cancer Hospital, Chinese Academy of Medical Sciences (CAMS) and Peking Union Medical College (PUMC) Beijing China; ^4^ Department of Oncology, National Cancer Center/National Clinical Research Center for Cancer/Cancer Hospital, Chinese Academy of Medical Sciences (CAMS) and Peking Union Medical College (PUMC) Beijing China; ^5^ Moffitt Cancer Center Tampa Florida USA; ^6^ Morsani College of Medicine University of South Florida Tampa Florida USA; ^7^ Division of Hepatology, Department of Medicine and Northwell Center for Liver Diseases & Transplantation Northwell Health Manhasset New York USA; ^8^ Department of General Surgery Tan Tock Seng Hospital Singapore Singapore; ^9^ Department of Gastrointestinal Oncology Moffitt Cancer Center Tampa Florida USA; ^10^ Division of Hematology and Oncology, Perlmutter Cancer Center of NYU Langone Health, NYU Grossman School of Medicine New York New York USA

**Keywords:** hepatocellular carcinoma (HCC), prognosis, radiotherapy, regorafenib, second‐line regimen

## Abstract

**Purpose:**

This study aimed to assess the efficacy and safety of intensity‐modulated radiotherapy (IMRT) combined with regorafenib with or without immune checkpoint inhibitors (ICIs) as a second‐ or later‐line treatment for advanced hepatocellular carcinoma (HCC).

**Materials and Methods:**

Patients diagnosed with advanced HCC who had received RT combined with concurrent or sequential regorafenib treatment or regorafenib plus ICIs after failures of at least one line of systemic treatment in a single center from April 2018 to August 2022 were retrospectively reviewed. Progression‐free survival (PFS) was the primary endpoint, while overall survival (OS), objective response rate (ORR), disease control rate (DCR), and toxicity were the secondary endpoints.

**Results:**

Fifty patients were included, with 44 (88.0%) in BCLC stage C, 37 (74.0%) having portal vein tumor thrombosis (PVTT), and 12 (24.0%) with extrahepatic metastasis. Thirty‐eight patients received conventional fractionated RT (56.4Gy/22–28f), while 12 received hyperfractionated RT (50Gy/5–10f). Twenty‐six were treated concurrently with regorafenib and 24 sequentially. ICIs were applied in 34 patients. For the entire cohort, when measured from the start of RT initiation, the median PFS and OS were 10.9 months and not reached. The corresponding 2‐year PFS and OS rates were 25.3% and 53.5%, respectively. When assessed from regorafenib initiation, the median PFS and OS were 5.9 months and not reached, with 2‐year PFS and OS rates of 22.8% and 54.9%, respectively. For tumors in the RT field, the ORR was 74.0% (RECIST) and 92.0% (mRECIST). The most common grade 3 toxicities were hand‐foot syndrome (16.0%), thrombocytopenia (8.0%), dermatitis (8.0%), and transaminase elevation (6.0%).

**Conclusion:**

IMRT concurrently or sequentially combined with regorafenib with or without ICIs is an effective, well‐tolerated, and promising regimen as second‐line or further‐line treatment in patients with advanced HCC.

## 1 Introduction

Hepatocellular carcinoma (HCC) is the sixth most common cancer and the third leading cause of cancer mortality worldwide [1]. According to Barcelona Clinic Liver Cancer (BCLC) staging and treatment strategy of 2022, surgical resection, ablation, transplant, and transarterial chemoembolization (TACE) are the options for first‐line treatments in patients with BCLC 0‐B stage HCC with preserved liver function, while systematic therapy with tyrosine kinase inhibitors (Lenvatinib/sorafenib), atezolizumab plus bevacizumab and durvalumab plus tremelimumab is recommended as the first‐line strategy for those with BCLC C stage HCC [2, 3, 4]. However, the median progression‐free survival (PFS) observed with these regimens ranged from 4.3 to 7.4 months, and the overall response rate (ORR) was between 24.1% and 27.3% [3, 4].

Regorafenib is an oral multikinase inhibitor that has been shown to have antiangiogenic, antitumorigenic, and even anti‐metastatic effects [5]. In a phase 3 study (RESORCE study), regorafenib improved PFS (median PFS 3.1 months) over placebo (median PFS 1.5 months) for patients who progressed during sorafenib treatment [6]. Thus, regorafenib was recommended as a second‐line option in managing advanced HCC. However, the PFS and OS for patients with HCC with second‐line systemic therapies were still poor even when programmed cell death protein 1 (PD‐1) or programmed death‐ligand 1 (PD‐L1) was applied with antiangiogenic therapy. A previous study confirmed that regorafenib combined with immunotherapy, as compared with regorafenib alone, may significantly prolong OS (12.9 vs. 10.3 months; *p* = 0.010) and PFS (5.9 vs. 3.0 months; *p* < 0.001) as a second‐line therapy [7]. Another study reported that regorafenib combined with PD‐1 yielded an OS of 13.7 months and a PFS of 4.0 months [8]. In this situation, local therapies should be considered in combination with systemic therapies to improve the survival of these patients.

Several studies have explored the efficacy and safety of IMRT for advanced HCC. With tolerable toxicity, the ORR and survival of patients with HCC after RT were promising and encouraging [9]. Combinations of locoregional therapy with systemic therapy have been further investigated and shown to result in a meaningful survival improvement for patients with advanced HCC with preserved liver function [10]. Preclinical studies have reported that RT combined with targeted therapy with or without immunotherapy exerts synergistic antitumor activity [10, 11]. However, studies examining RT for advanced HCC as a second‐line therapy are ongoing, and the efficacy and safety of RT combined with regorafenib with or without ICIs as a second‐line therapy have yet to be investigated.

This real‐world study aimed to assess the efficacy and safety of RT combined with regorafenib with or without ICIs as a second or later‐line treatment for advanced HCC.

## 2 Material and Methods

### 2.1 Study Populations

Patients diagnosed with advanced HCC were retrospectively reviewed at the National Cancer Center/Chinese Academy of Medical Sciences and Peking Union Medical College, Beijing, China, between April 2018 and August 2022. The eligibility criteria were the following: patients with image‐based diagnosis or pathological diagnosis of HCC [12]; patients who received RT combined with concurrent regorafenib as a second‐line therapy after failure of first‐line systemic therapy or those who received sequential regorafenib as a later‐line therapy after the failure of first‐line systematic therapy followed by RT (A. Patients who were not suitable for surgery (especially with PVTT) with preserved liver functions and enough liver volume (usually more than 700 mL) would be eligible to undergo RT; B. RT could be applied to intrahepatic lesions); patients treated with anti‐PD‐1 or anti‐PD‐L1 ICIs concurrently with regorafenib in those at high risk of distant metastasis/with distant metastasis or for those in whom the efficacy of regorafenib was unsatisfactory (high risks of distant metastasis included out‐field lesions, lymph node metastasis, distant metastasis, and major vascular involvements); patients with preserved liver function (alanine transaminase/aspartate transaminase/creatinine (ALT/AST/Cr) < 2‐fold upper limits, Child‐Pugh grade A‐B, and ALBI grade 1–2) who had vascular invasion, lymphatic metastasis, or extrahepatic metastasis; and patients received liver lesion‐directed RT. In concurrent regorafenib, the timing of RT administration within treatment course was at the previous first‐line or second‐line systematic treatment failure. In sequential regorafenib group, the timing of RT was after the locaregional therapies failure (including surgery, ablation, and TACE etc.). Patients were excluded if they underwent previous RT and regorafenib therapy. Figure S1 showed schematic treatment diagram of enrolled patients. This study was approved by the ethics committee or institutional review board (approval number: 22/094–3295) and complied with good clinical practice guidelines, the Declaration of Helsinki [13].

### 2.2 Radiotherapy

In a fasting state, patients received 100 mL oral contrast agent half an hour before simulation to indicate the duodenum and intestine. Patients were immobilized with a thermoplastic body membrane in supine position with both arms above the forehead and the right hand over the left. Contrast computed tomography (CT), 4‐dimensional computed tomography (4DCT), and dynamic enhanced magnetic resonance (MR) simulations were performed under free breathing. Image registration was accomplished in the Pinnacle treatment planning system (Philips Healthcare, Amsterdam, The Netherlands).

Based on the CT and MR simulation images, the gross tumor volume (GTV) was delineated to encompass the primary hepatic lesions, as well as any tumor thrombus and lymph node metastasis present in the patients. The clinical target volume (CTV) was expanded 5 mm from the GTV in all directions, 10–15 mm in the direction of blood vessels with tumor thrombus, and lymph node drainage region if regional lymph node metastasis was available. The planning tumor volume (PTV) was expanded according to 4D‐CT, usually 5 mm in the anterior–posterior and left–right directions and 10–15 mm in the superior–inferior direction. The prescription dose to the GTV that was dependent on the surrounding organs at risk (OARs) was planned at 50–70 Gy in 20–30 fractions or 30–50 Gy in 5–10 fractions. The prescription dose to the PTV was planned at 40–50 Gy in 20–30 fractions or 25–40 Gy in 5–10 fractions.

The OARs were also delineated, including the stomach, duodenum, intestine, colon, whole liver, spinal cord, and kidneys. The dose‐volume constraints for OARs were formulated based on the fractionation regimens [14]. The dose constraints for the OARs in conventional fractionation were as follows: whole liver minus GTV, mean dose (Dmean) ≤ 24 Gy; stomach, duodenum, and intestine, maximum dose (Dmax) ≤ 54 Gy and V50 ≤ 10 mL; colon, Dmax ≤ 55 Gy and V52 ≤ 10 mL; spinal cord planning risk volume (PRV), Dmax ≤ 40 Gy; and left and right kidney, V20 ≤ 30%. All patients were treated with IMRT or volumetric‐modulated arc therapy (VMAT) technology and underwent cone beam CT (CBCT) scanning at a certain frequency before beam delivery.

### 2.3 Systemic Treatment

All patients received RT combined with concurrent or sequential regorafenib or regorafenib plus anti‐PD‐1 or anti‐PD‐L1 as a second‐line or later‐line therapy. Concurrent regorafenib was given at the start date of RT. Sequential regorafenib was given until out‐field or distant progressions were observed after RT therapy. Patients included in our study were poorly tolerant to second‐line systemic therapy, and concurrent RT might further decrease their tolerance. Therefore, according to ECOG score, previous tolerance to systemic therapy, nutritional status, etc., all patients began with a regorafenib dose of 80–120 mg per day, which was increased to at least 120 mg daily if well‐tolerated. If serious toxicities were observed, the dose would be reduced. According to the multidisciplinary consultation, ICIs could be administered concurrently with regorafenib to patients at high risk of distant metastasis or with distant metastasis or to those for whom the efficacy of regorafenib was unsatisfactory by MDT members. ICIs could be given at the start date of regorafenib or during the regorafenib administration. In terms of its sequence with RT, ICIs could be given either during the RT procedure or after RT. Laboratory tests were conducted before ICI therapy and included blood routine, urine routine, blood chemistry, thyroid function, adrenal/pituitary function, and pancreatic amylase and lipase. Anti‐hepatitis B virus (HBV) therapy was administrated for patients with detectable HBV copies.

### 2.4 Follow‐Up and Evaluation

Imaging examinations (including abdominal MR and chest CT) and laboratory tests (including blood routine, blood biochemistry, urine routine, and serum AFP) were performed in the first month and third month after RT and every 3 months thereafter. The clinical outcomes were evaluated using the Response Evaluation Criteria in Solid Tumors version (RECIST) v. 1.1 [15] and the modified RECIST (mRECIST) [16].

PFS (calculated from the date of RT until the date of regorafenib failure or death) was the primary endpoint. OS was calculated from the date of RT until the date of death or the last follow‐up appointment. However, defining endpoints from the start of RT may introduce a potential bias in the sequential group. To eliminate this, PFS and OS were also calculated from the initiation of regorafenib. In alignment with the established methodology for defining PFS1 and PFS2 in studies pertaining to first‐line and second‐line therapies within the field of systemic therapy, we have also established dual PFS endpoints for the sequential regorafenib group. PFS1 (calculated from RT initiation to RT failure) and PFS2 (calculated from regorafenib initiation to regorafenib treatment failure or death) were recorded in the sequential regorafenib group.

ORR [defined as complete response (CR) + partial response (PR)], disease control rate [DCR; defined as CR + PR + stable disease (SD)], and toxicity were the secondary endpoints. The toxicity was evaluated using the Common Terminology Criteria for Adverse Events (CTCAE) v. 5.0.

### 2.5 Statistical Analysis

Baseline characteristics were examined using descriptive analysis. OS and PFS were estimated using the Kaplan–Meier Method, while differences between subgroups were assessed with the log‐rank test. Univariate and multivariate analyses were performed to identify the variates (including age, AFP level, BCLC stage, ALBI grade, concurrent/sequential regorafenib therapy, extrahepatic metastasis, lung metastasis, vascular invasion, ICI therapy, and the number of lesions) associated with unfavorable clinical outcomes. Certain variables demonstrated clinical relevance to the outcomes, yet they failed to reach statistical significance in the univariate analysis. To avoid potential interactions among variables and to ensure the inclusion of all clinically significant variables, we incorporated all covariates into the multivariate model. All statistical analyses were performed using SPSS v. 26 (IBM Corp., Armonk, NY, USA) and R v. 3.5.1 (The R Foundation for Statistical Computing, Vienna, Austria). A 2‐sided *p* value < 0.05 was indicative of a statistically significant difference.

## 3 Results

### 3.1 Patient Characteristics

A total of 54 patients diagnosed with advanced HCC were enrolled. Two patients received radiotherapy for bone metastasis and two for lung metastasis without liver lesions. Ultimately, a total of 50 patients were included in the analysis. The baseline characteristics are shown in Table 1. The treatment characteristics are shown in Table 2. The median age was 55 (range, 31–73) years, and 47 (94.0%) patients were male. Some patients progressed to the advanced stage after local therapy. This included 2 patients (4.0%) with a history of hepatectomy, 16 patients (32.0%) with a history of TACE, 5 patients (10.0%) who underwent both surgical resection and TACE, 7 patients (14.0%) with a combination of ablation and TACE, and 3 patients (6.0%) who had previously undergone surgical resection, ablation, and TACE. Previous systemic treatments included sorafenib (*n* = 19), lenvatinib (*n* = 13), PD‐1 alone (*n* = 2), TKIs + TKIs (*n* = 7), and TKIs + ICIs (*n* = 9). Forty‐four (88.0%) patients had BCLC C stage. Portal vein tumor thrombosis (PVTT) was observed in 37 (74.0%) patients, extrahepatic metastasis in 12 (24.0%) patients, and lung metastasis in 9 (18.0%) patients. The median size of the primary tumor was 9.7 cm (range, 1.0–22.6 cm), and 29 (58.0%) patients had multiple hepatic lesions. Additionally, 35 (70.0%) patients were classified as ALBI grade 1 and 49 (98.0%) as Child‐Pugh A, with 45 (90.0%) having an A5 score. The median value of AFP was 1915.5 ng/mL (range, 1.91–484,000 ng/mL). Moreover, all patients were infected with HBV; 26 (52.0%) patients were treated with RT with concurrent regorafenib and 24 (48.0%) with sequential regorafenib. The median dose of conventional fractionation RT was 56.4 Gy (50–69.8 Gy) in 22–28 fractions and 50 Gy (30–54 Gy) in 5–10 fractions for hyperfractionation RT. Anti‐PD‐1/anti‐PD‐L1 agents were applied in 34 (68.0%) patients treated with regorafenib and included camrelizumab, sintilimab, tislelizumab, and toripalimab as anti‐PD‐1 therapy and durvalumab as the anti‐PD‐L1 therapy (Table 2). The distribution of patients across the different ICIs regimens was roughly balanced between the concurrent regorafenib group and the sequential regorafenib group. The median dose of regorafenib was 120 mg (range, 80‐160 mg) per day. The median durations of regorafenib treatment were 6.7 months and 5.4 months in the concurrent regorafenib group and the sequential regorafenib group.

**TABLE 1:** Demographic and clinical characteristics of the patients who received radiotherapy and regorafenib with or without immunotherapy before RT.

Variables	N (%)	Variables	N (%)
Age (years)	55.0	Tumor size (cm)	9.7
median (range)	(31.0–73.0)	median (range)	(1.0–22.6)
Sex		BCLC stage at RT administration^a^	
Male	47 (94.0)	A	3 (6.0)
Female	3 (6.0)	B	3 (6.0)
ECOG score		C	44 (88.0)
0	14 (28.0)	Lymph node metastasis	
1	35 (70.0)	No	38 (76.0)
2	1 (2.0)	Yes	12 (24.0)
Cirrhosis		Distant metastasis	
No	23 (46.0)	No	38 (76.0)
Yes	27 (54.0)	Yes	12 (24.0)
ALBI score, mean (SD)	−2.76 (0.45)	Lung metastasis	
ALBI grade		No	41 (82.0)
Grade 1	35 (70.0)	Yes	9 (18.0)
Grade 2	15 (30.0)	PVTT	
Child‐Pugh classification		No	13 (26.0)
A5	45 (90.0)	Vp2	5 (10.0)
A6	4 (8.0)	Vp3	18 (36.0)
B7	1 (2.0)	Vp4	14 (28.0)
AFP level (ng/mL) median (range)	1915.5 (1.91–484,000.0)	AJCC (8th) stage at RT administration^b^	
Etiology		I	2 (4.0)
HBV	50 (100.0)	II	1 (2.0)
Number of lesions		III	26 (52.0)
Single	21 (42.0)	IV^c^	21 (42.0)
Multiple	29 (58.0)		

Abbreviations: AFP, alpha‐fetoprotein; AJCC, American Joint Committee on Cancer; ALBI, albumin‐bilirubin; BCLC, Barcelona Clinic Liver Cancer; ECOG, Eastern Cooperative Oncology Group; HBV, hepatitis B virus; LNM, Lymph node metastasis; PVTT, portal vein tumor thrombus.

^a^ Six patients were staged as BCLC stage A/B at RT administration. However, they experienced disease progression after RT. Following RT progression, they received sequential regorafenib treatment. Consequently, the BCLC stage of these six patients escalated to stage C at the time of regorafenib administration.

^b^ Three patients were staged as AJCC I/II at RT administration. They experienced disease progression after RT. These three patients escalated to AJCC stage IV at the time of regorafenib administration.

^c^ There were six enrolled patients with both LNM and distant metastasis.

**TABLE 2:** Treatment characteristics of the patients who received radiotherapy and regorafenib with or without immunotherapy.

Variables	N (%)
Previous systemic therapy	
First‐line therapy	42 (84.0)
≥ Second‐line therapy	8 (16.0)
Previous systemic regimens	
Sorafenib	19 (38.0)
Lenvatinib	13 (26.0)
Toripalimab	2 (4.0)
Sorafenib + lenvatinib	6 (12.0)
Sorafenib + apatinib	1 (2.0)
Sintilimab+IBI305	1 (2.0)
Lenvatinib + toripalimab	2 (4.0)
Lenvatinib + tislelizumab	1 (2.0)
Lenvatinib + sintilimab	2 (4.0)
Lenvatinib + pembrolizumab	1 (2.0)
Lenvatinib + camrelizumab	1 (2.0)
Apatinib + camrelizumab	1 (2.0)
Previous locoregional treatment	
Surgical resection	2 (4.0)
TACE	16 (32.0)
Surgical resection + TACE	5 (10.0)
Ablation + TACE	7 (14.0)
Surgical resection + Ablation + TACE	3 (6.0)
Prescription dose (Gy), median (range)	57.4 (30–69.8)
ICIs therapy	
No	16 (32.0)
Yes	34 (68.0)
ICI regimens in concurrent regorafenib group	
PD‐1	2 (4.0)
Camrelizumab	3 (6.0)
Tislelizumab	3 (6.0)
Toripalimab	4 (8.0)
Sintilimab	3 (6.0)
ICI regimens in sequential regorafenib group	
PD‐1	3 (6.0)
Camrelizumab	2 (4.0)
Tislelizumab	3 (6.0)
Toripalimab	5 (10.0)
Sintilimab	5 (10.0)
Durvalumab	1 (2.0)
Sequence of RT and regorafenib	
Sequential regorafenib	24 (48.0)
Concurrent regorafenib	26 (52.0)

Abbreviations: ICI, immune checkpoint inhibitor; RT, radiotherapy; TACE, transarterial chemoembolization.

### 3.2 Treatment Response

The best treatment response at 3 months after RT is shown in Table 3. For tumors in the field of RT, the ORR was 74.0% and 92.0% according to the RECIST and mRECIST criteria, respectively. The ORR was 36.0% and 46.0% for all lesions according to the RECIST and mRECIST criteria, respectively.

**TABLE 3:** Best treatment response evaluation at 3 months after RT.

Response	In field of RT		Total body	
	RECIST, *n* (%)	mRECIST, *n* (%)	RECIST, *n* (%)	mRECIST, *n* (%)
CR	3 (6.0)	11 (22.0)	1 (2.0)	5 (10.0)
PR	34 (68.0)	35 (70.0)	17 (34.0)	18 (36.0)
SD	13 (26.0)	4 (8.0)	8 (16.0)	3 (6.0)
PD	0 (0.0)	0 (0.0)	24 (48.0)	24 (48.0)
ORR	37 (74.0)	46 (92.0)	18 (36.0)	23 (46.0)
DCR	50 (100.0)	50 (100.0)	26 (52.0)	26 (52.0)

Abbreviations: CR, complete response; DCR, disease control rate; mRECIST, modified Response Evaluation Criteria in Solid Tumors; ORR, objective response rate; PD, progressive disease; PR, partial response; RECIST, Response Evaluation Criteria in Solid Tumors; RT, radiotherapy; SD, stable disease.

### 3.3 Survival and Prognostic Factors

After a median follow‐up of 18.2 months (95% CI: 15.9–37.4), 13 patients died, and 42 patients progressed. Four patients were lost to follow‐up, who were in critical condition at the time of their last follow‐up. Follow‐up was conducted via telephone, but the patient's family declined to share details regarding the medical condition. However, these four patients continued to be followed up for a period ranging from 8 to 27 months.

#### 3.3.1 Survival Outcomes (Measured From RT Initiation)

**FIGURE 1:**
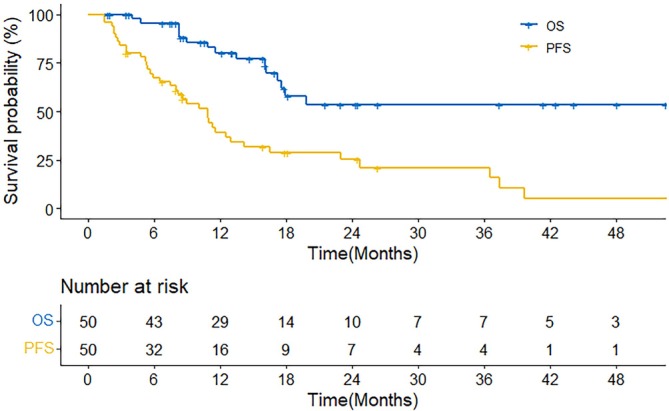
OS and PFS of patients with advanced HCC who had received RT combined with concurrent or sequential regorafenib treatment or regorafenib plus anti‐PD‐1 or anti‐PD‐L1 ICIs, both PFS and OS measured from radiotherapy initiation. OS, overall survival; PFS, progression‐free survival; HCC, hepatocellular carcinoma; RT, radiotherapy; PD‐1, programmed cell death protein 1; PD‐L1, programmed death‐ligand 1; ICIs, immune checkpoint inhibitors.

For the entire cohort, the median PFS was 10.9 months (95% CI: 8.0–14.2), with a 1‐year PFS rate of 39.2% and a 2‐year PFS rate of 25.3% (Figure 1). The median OS was not reached, with a 1‐year OS rate of 80.1% and a 2‐year OS rate of 53.5% (Figure 1). And the landmark analysis results of PFS and OS of patients with advanced HCC were showed in Figure S2. The 1‐year OS rate and 2‐year OS rate were 80.1% and 53.5%.

**FIGURE 2:**
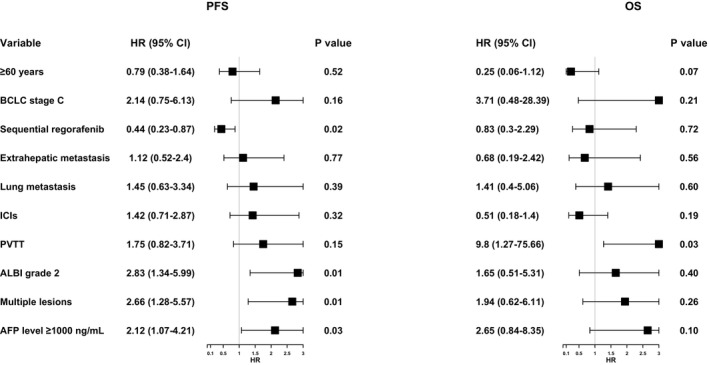
Univariate analysis for PFS and OS of patients with advanced HCC, both PFS and OS measured from radiotherapy initiation. PFS, progression‐free survival; OS, overall survival; HR, hazard ratio; CI, confidence interval; BCLC, Barcelona Clinic Liver Cancer; PVTT, portal vein tumor thrombus; ALBI, albumin‐bilirubin; HCC, hepatocellular carcinoma; AFP, alpha‐fetoprotein.

In univariate analysis (Figure 2), a significant improvement of PFS was observed in the ALBI grade 1 group as compared with the ALBI grade 2 group [median PFS: 12.5 vs. 7.5 months; hazard ratio (HR) 2.83, 95% CI: 1.34–5.99; *p* = 0.01] (Figure 2) and in the single‐lesion group compared with the multiple‐lesion group (median PFS: 14.2 vs. 8.2 months; HR 2.66, 95% CI: 1.28–5.57; *p* = 0.01) (Figure 2). There was also a significant difference in PFS between patients with AFP < 1000 ng/mL group and those with AFP ≥ 1000 ng/mL group (13.0 vs. 8.0 months; HR 2.12, 95% CI: 1.07–4.21; *p* = 0.03) (Figure 2). But no significant difference in PFS was observed between patients with PVTT and those without PVTT. But for OS, only PVTT was the significant prognostic factor (17.6 months with PVTT versus not reached without PVTT; HR 9.80, 95% CI: 1.27–75.66; *p =* 0.03) (Figure 2).

The variables included in the final multivariate model were age, BCLC stage, the sequence of regorafenib treatment, extrahepatic metastasis, lung metastasis, immunotherapy, PVTT, ALBI grade, number of lesions, and AFP level. The results showed that sequential regorafenib was the prognostic factor for favorable PFS. In contrast, ALBI grade 2, multiple lesions, and AFP level ≥ 1000 ng/mL were the risk factors for shorter PFS. In addition, PVTT was an independent poor prognostic factor for poor OS.

#### 3.3.2 Survival Outcomes (Measured From Regorafenib Initiation)

After controlling the immortal bias in patients receiving sequential regorafenib, for the entire cohort, the median PFS was 5.9 months (95% CI: 4.2–13.9), with a 1‐year PFS rate of 33.3% and a 2‐year PFS rate of 22.8%. The median OS was not reached; the corresponding 1‐year and 2‐year OS rates were 68.3% and 54.9%. For the concurrent regorafenib group, the median PFS was 5.9 months (95% CI: 3.5‐NA), with 1‐year and 2‐year OS rates of 73.1% and 54.8%, respectively. For the sequential regorafenib group, the PFS1, defined as the interval from the start of RT to regorafenib initiation upon RT failure, was 5.9 months (95% CI: 4.4–7.8). For the sequential regorafenib group, the median PFS2 (calculated from regorafenib initiation) was 5.4 months (95% CI: 4.4‐NA), with the 1‐year and 2‐year OS rates of 62.6% and 54.7%, respectively (Figure S3). There were no differences between the concurrent regorafenib group with RT and the sequential regorafenib group after RT progression in PFS (5.9 vs. 5.4 months; HR 0.95, 95% CI: 0.50–1.82; *p* = 0.879) and OS (HR 1.31, 95% CI: 0.47–3.63; *p* = 0.592).

### 3.4 Failure Patterns

No progression was observed in the field of RT. Intrahepatic progression out of the field of RT and distance metastasis were the main failure patterns. Intrahepatic progression out of the field of RT was found in 16 patients and distance metastasis in 17 patients. Both intrahepatic progressions out of the field of RT and distance metastasis were found in 9 patients.

### 3.5 Adverse Event

The toxicity profiles related to treatment are shown in Table 4. Patients tolerated treatment well. The most common grade 3 toxicities were hand‐foot syndrome (16.0%), thrombocytopenia (8.0%), dermatitis (8.0%) and transaminase elevation (6.0%). There was no significant difference in grade 3 toxicities between the concurrent regorafenib group and the sequential regorafenib group. Classical or non‐classical radiation‐induced liver disease [17] was not observed. Among the 34 patients who underwent immunotherapy, no instances of fatal immune‐related toxicity were observed.

**TABLE 4:** Adverse event.

Adverse events	Any grade	G1	G2	G3
Dermatitis	24 (48.0)	13 (26.0)	7 (14.0)	4 (8.0)*
Radiation esophagitis	2 (4.0)	0	2 (4.0)	0
Transaminase elevation	12 (24.0)	7 (14.0)	2 (4.0)	3 (6.0)*
Blood bilirubin increase	9 (18.0)	6 (12.0)	3 (6.0)	0
Hypokalemia	1 (2.0)	0	1 (2.0)	0
Hypoalbuminemia	10 (20.0)	9 (18.0)	1 (2.0)	0
Anemia	4 (8.0)	4 (8.0)	0	0
Leukopenia	32 (64.0)	13 (26.0)	17 (34.0)	2 (4.0)
Neutropenia	4 (8.0)	3 (6.0)	1 (2.0)	0
Thrombocytopenia	20 (40.0)	7 (14.0)	9 (18.0)	4 (8.0)*
Fever	6 (12.0)	4 (8.0)	2 (4.0)	0
Ascites	4 (8.0)	2 (4.0)	2 (4.0)	0
Fatigue	25 (50.0)	10 (20.0)	14 (28.0)	1 (2.0)
Anorexia	28 (56.0)	22 (44.0)	6 (12.0)	0
Hand‐foot syndrome	18 (36.0)	4 (8.0)	6 (12.0)	8 (16.0)*
Pain	11 (22.0)	2 (4.0)	7 (14.0)	2 (4.0)
Diarrhea	11 (22.0)	5 (10.0)	5 (10.0)	1 (2.0)
Abdominal distension	6 (12.0)	4 (8.0)	2 (4.0)	0
Nausea	11 (22.0)	8 (16.0)	3 (6.0)	0
Vomiting	2 (4.0)	1 (2.0)	1 (2.0)	0
Hypertension	6 (12.0)	1 (2.0)	3 (6.0)	2 (4.0)
Bleeding	4 (8.0)	3 (6.0)	1 (2.0)	0

*Note:* Data are shown as *n* (%). G, grade.

## 4 Discussion

To our knowledge, this is the first real‐world retrospective study evaluating the efficacy of RT combined with regorafenib with or without ICIs as second‐line or further‐line therapy for HCC. Most enrolled patients were at an advanced stage, including 44 (88.0%) at BCLC stage C, with 37 (74.0%) having PVTT and 12 (24.0%) having distant metastasis; the median lesion size was 9.7 cm. Despite these conditions, when measured from the start of RT initiation, favorable survival outcomes were obtained (median PFS of 10.9 months and 2‐year OS rate of 53.5%). Even when measured from the initiation of regorafenib, favorable PFS and OS outcomes were achieved, with a median PFS of 5.9 months and a 2‐year OS of 54.9%. Thus, this treatment could be an effective and encouraging regimen with manageable toxicity in advanced patients with HCC.

The outcomes in our study were promising, and superior to survivals of other recommended second‐line regimens. The second‐line systemic regimens that have been approved for treating advanced patients with HCC after sorafenib failure include regorafenib, cabozantinib, and ramucirumab [18]. A phase 3 trial (RESORCE trial) reported that regorafenib, compared with placebo, improved the median OS (10.6 vs. 7.8 months; *p* < 0.0001), median PFS (3.1 vs. 1.5 months; *p* < 0.0001), and ORR (11.0% vs. 4.0%; *p* = 0.0047) in patients who experienced disease progression during sorafenib treatment [6]. Similarly, in the real‐world REFINE trial [19], regorafenib yielded a median PFS of 3.9 months and a median OS of 11.1–15.8 months. Other drugs tested in phase 3 trials, including cabozantinib in the CELESTIAL trial [20] and ramucirumab in REACH trial [21] were associated with improved OS (cabozantinib: 10.2 vs. 8.0 months, *p* = 0.005; ramucirumab: 9.2 vs. 7.6 months, *p* = 0.14) and PFS (cabozantinib: 5.2 vs. 1.9 months, *p* < 0.001; ramucirumab: 2.8 versus 2.1 months, *p* < 0.001) as compared with placebo. The optional second‐line regimens were various [22, 23, 24, 25, 26, 27, 28], including sorafenib, regorafenib, cabozantinib, lenvatinib, and ICIs, etc. Regardless the first‐line systemic regimen, the PFS of second‐line regimens varied from 1.8 months to 5.4 months (Table S1). However, the survival benefit from second‐line systemic treatment for patients with advanced HCC reported in these trials is modest at best. Data from our real‐world study has a small sample size and is not powered to confirm OS or PFS benefit in patients with advanced HCC who experienced failure of sorafenib or lenvatinib. The promising results, however, suggest that locoregional therapy added to systemic therapy can potentially provide positive clinical outcomes.

Furthermore, in the sequential treatment group, the favorable control of challenging conditions such as tumor thrombus by prior RT significantly extended both PFS and OS following the use of regorafenib after RT failure. Therefore, we believe that reporting both PFS1 and PFS2 here offers a more reasonable interpretation of clinical observations.

Additionally, the survival benefits after regorafenib plus RT with or without ICIs for more advanced HCC patients were potentially promising. At present, for patients with HCC initially diagnosed at an advanced stage or those reaching advanced stage after failure of locoregional therapy, the first‐line treatment recommendation from the European Association for the Study of the Liver (EASL) mainly centers on systemic therapy, which includes atezolizumab plus bevacizumab (IMbrave150 trial) or durvalumab plus tremelimumab (HIMALAYA trial) followed by lenvatinib (REFLECT trial), durvalumab (HIMALAYA trial), sorafenib (SHARP trial) [2, 4, 6, 29, 30, 31] In the IMbrave150 trial, the median OS was 19.2 months in the atezolizumab plus bevacizumab group after an extensive long follow‐up period [31]; meanwhile, in the HIMALAYA trial, the median OS was 16.4 months in the tremelimumab plus durvalumab group. Lenvatinib and durvalumab were shown not inferior to sorafenib according to the REFLECT trial (median OS: 13.6 vs. 12.3 months) and HIMALAYA trial (median OS: 16.6 vs. 13.8 months) [4, 30]. Additionally, other trials such as Leap‐002 [32], ORIENT‐32 [33], and RESCUE [34] also produced satisfactory outcomes, the most promising of which was from the Leap‐002 trial, where a regimen of lenvatinib plus pembrolizumab achieved prolonged survival outcomes as compared with lenvatinib alone (median OS: 21.2 vs. 19.0 months; median PFS: 8.1 vs. 8.0 months). Our study with a 2‐year OS rate of 53.5%, although achieved with a small sample size in a real‐world study, indicates that the similar approach of combining systemic therapy along with RT in HCC could be potentially promising.

**FIGURE 3:**
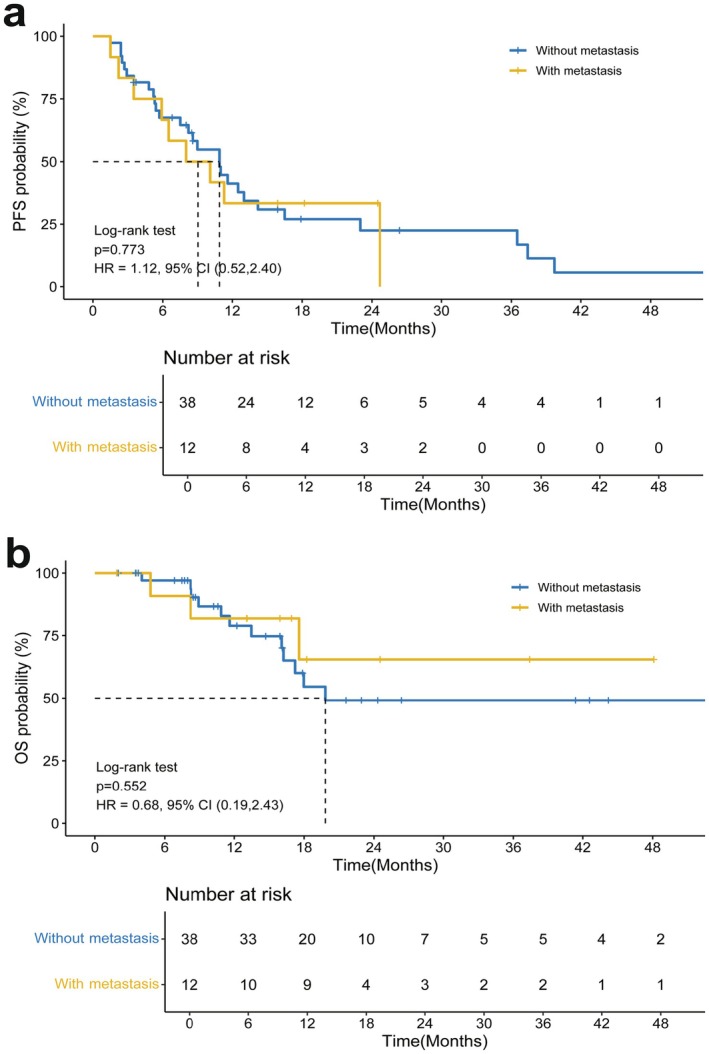
Subgroup analysis of the OS and PFS of patients with advanced HCC, both PFS and OS measured from radiotherapy initiation. (A) PFS of patients with distant metastasis versus those without distant metastasis. (B) OS of patients with distant metastasis versus those without distant metastasis. PFS, progression‐free survival; OS, overall survival; HR, hazard ratio; CI, confidence interval; HCC, hepatocellular carcinoma.

Our study demonstrated OS and PFS benefits regardless of distant metastasis, indicating that the survival‐threatening factors could be obviated by adding local RT (Figure 3). Figure S5,S6 show 2 cases with PVTT and primary tumor progression after first‐line treatment that were treated with RT concurrent with regorafenib. PVTT is sensitive to RT, and these cases demonstrated CR after RT according to mRECIST criteria. PVTT poses the greatest risk to patients' lives—even more so than lung metastasis—and RT was able to prolong survival even after PVTT occurred. Thus, obvious improvements in OS in patients with distant metastasis were observed. With a similar molecular structure to sorafenib but greater antitumor activity, regorafenib is an oral tyrosine kinase inhibitor that can target VEGFR 1–3, tyrosine receptor protein kinase TIE, RET, PDGFR, FGFR, and RAF for advanced HCC after sorafenib failure [18, 35]. The VEGF pathway promotes local immune suppression through the inhibition of antigen‐presenting cells and effector cells as well as through the activation of suppressive elements, including T regulatory cells, myeloid‐derived suppressor cells, and tumor‐associated macrophages; this constitutes the rationale for combining ICIs with antiangiogenic agents [36]. Some clinical data have shown encouraging outcomes for targeted therapy in combination with ICI therapy. Based on this, RT combined with regorafenib plus ICI therapy demonstrated a favorable survival benefit even in patients with poor prognosis.

Multivariate analysis demonstrated that ALBI grade ≥ 2, multiple lesions, and AFP level ≥ 1000 ng/mL were poor prognostic factors for PFS (shown in Figure 2, Figure S4). Therefore, we suggest that a second‐line therapy including RT plus targeted therapy with immunotherapy should be conducted as early as possible when the first‐line therapy fails.

**FIGURE 4:**
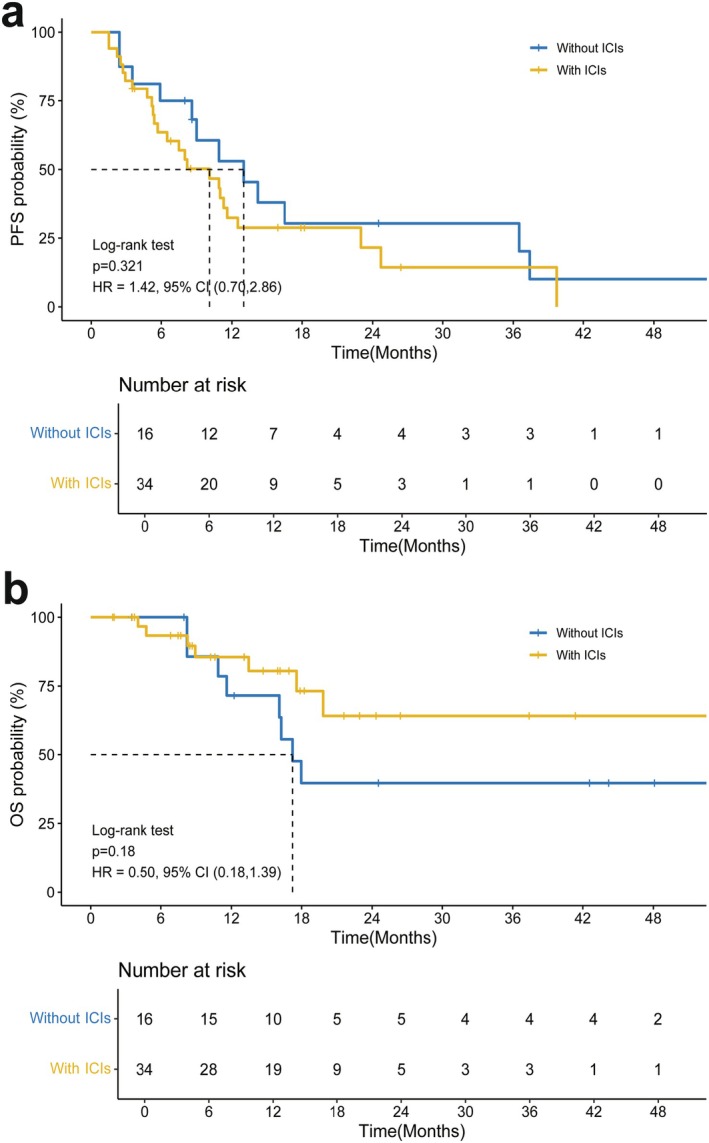
Subgroup analysis of the OS and PFS of patients with advanced HCC, both PFS and OS measured from radiotherapy initiation. (A) PFS of patients with ICI treatment versus without ICI treatment. (B) OS of patients with ICI treatment versus without ICI treatment. PFS, progression‐free survival; OS, overall survival; HR, hazard ratio; CI, confidence interval; ICI, immune checkpoint inhibitor; HCC, hepatocellular carcinoma.

**FIGURE 5:**
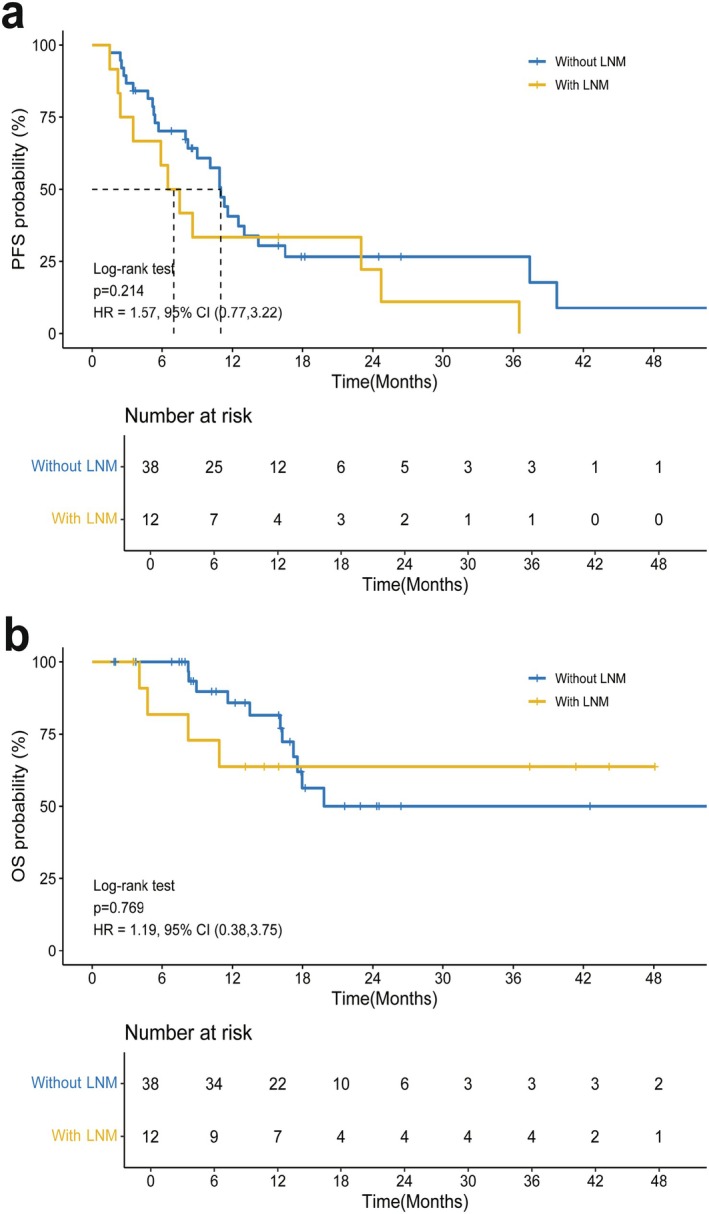
Subgroup analysis of the OS and PFS of patients with advanced HCC, both PFS and OS measured from radiotherapy initiation. (A) PFS of patients with LNM versus without LNM. (B) OS of patients with LNM versus without LNM. PFS, progression‐free survival; OS, overall survival; HR, hazard ratio; CI, confidence interval; LNM, lymph node metastasis; HCC, hepatocellular carcinoma.

There was no significant difference in OS and PFS between the ICI and non‐ICI groups (Figure 4). There was also no significant difference in OS and PFS between patients with lymph node metastasis and those without lymph node metastasis (Figure 5). Patients with hepatic, lymph node metastasis, and distant metastasis had a poor prognosis, which could be reversed by ICI treatment. As a result, those with poor prognosis factors may obtain similar outcomes to those without hepatic, LNM, or distant metastasis. Interestingly, we also found that the response rate of regorafenib plus ICI could translate into a long‐term effect, even 20 months after treatment.

With the improvement of RT technology, RT is considered to be an effective and safe option among patients with advanced HCC. IMRT and VMAT technology have been shown to spare the liver tissue from radiation damage. Previous studies have reported that HCC is sensitive to RT, and combinations of RT and targeted therapies may positively influence the survival outcomes of those with advanced HCC. A prospective phase 2 study from China demonstrated that sorafenib in combination with RT was an effective, well‐tolerated, and promising treatment for patients with HCC with portal vein and/or hepatic vein invasion, yielding a median OS and time to progression of 16.8 months and 7.1 months, respectively [37]. Another study also confirmed the efficacy of RT and sorafenib combination therapy in advanced HCC [38]. Together, these findings point to the synergistic effect of combined therapy in treating advanced HCC. Under similar therapy regimes, RT involvement may still be of value for patients with advanced HCC receiving a second‐line or further‐line systematic therapy.

Table 4 showed the cumulative toxicity risks associated with the combination of regorafenib, ICIs, and radiotherapy. Dermatitis predominantly occurred within the radiation field. Concurrent regorafenib was associated with an increased incidence and severity of radiation dermatitis. All grade 3 dermatitis (8.0%) were happened in the patients with the concurrent regorafenib. Radiation esophagitis was observed specifically in patients receiving RT for vena cava tumor thrombus. Transaminase elevation was predominantly related to regorafenib since this condition could be resolved upon discontinuation or dose reduction of regorafenib. Upon normalization of transaminase levels, patients were restarted on a reduced dose of regorafenib (40–80 mg per day). In addition, diarrhea (22.0%), hand‐foot syndrome (36.0%), and hypertension (12.0%) were mainly caused by regorafenib therapy, which were consistent with that from RESORCE trial. The management of hand‐foot syndrome included neurotrophic medications (e.g., vitamin B1, B12), application of amine/urea‐containing hand cream, and avoidance of cold stimuli. And antihypertensive drugs were used for hypertension. Thrombocytopenia (40.0%) was the most significant hematologic toxicity, primarily attributed to hypersplenism secondary to liver cirrhosis, with additional contributions from both administrate of regorafenib and radiotherapy. Thrombopoietin (TPO) might effectively increase platelet counts without compromising radiation dose delivery, though it required regorafenib dose modifications.

Despite the promising findings, some limitations to this study should be mentioned. Firstly, we employed a retrospective design with a limited number of samples; nonetheless, this study represents the largest sample of RT combined with regorafenib reported on thus far. Secondly, loss of follow‐up and selection bias in a retrospective study were inevitable. Thirdly, this study included an HBV dominant Asian patient population and might not be generalizable. Forth, the encouraging PFS and OS observed in our study must be interpreted in light of potential confounders, including prior treatments and concomitant ICI use, which may limit direct comparability with historical controls. Therefore, these survival outcomes should be validated in prospective studies. Finally, the lack of a control group limits our ability to draw definitive conclusions about the additional benefits of IMRT and ICIs compared with regorafenib alone for advanced HCC patients. The results should be confirmed and expanded upon using a larger sample of data in a prospective study design, with comparisons being made to other treatment strategies.

## 5 Conclusions

In this real‐world retrospective study, RT concurrently or sequentially combined with regorafenib with or without ICIs is an effective, well‐tolerated, and promising regimen as second‐line or further‐line in patients advanced with HCC. ALBI grade 2, multiple lesions, and AFP level ≥ 1000 ng/mL were poor prognostic factors. Considering the efficacy of radiotherapy for PVTT, it seems that patients with PVTT might benefit from a second‐line therapy including targeted therapy and RT when first‐line therapy fails. Additional investigation of RT combined with systemic therapy such as TKI and/or ICIs in the second‐line HCC is warranted.

## Author Contributions

B.C. designed the research and contributed to the study concept; B.C., X.B., and Y.L. provided the administrative support; F.Y., Y.S., W.Z. S.W., Y.L., Y.T., H.J., H.F., S.Q., N.L., X.B., and B.C. provided the study materials or patient data; L.X., Z.L., Y.Z., and L.W. were involved in data collection and analysis. All authors were involved in the manuscript writing and approved the final version of the manuscript.

## Funding

This work was supported by the Noncommunicable Chronic Diseases‐National Science and Technology Major Project (grant No. 2024ZD0520501), the Beijing Natural Science Foundation (grant No. L248057), the CAMS Innovation Fund for Medical Sciences (CIFMS) (grant No. 2024‐I2M‐C&T‐B‐067), and the National High Level Hospital Clinical Research Funding (grant No. 2025‐LYZX).

## Ethics Statement

This study was approved by the ethics committee or institutional review board (approval number: 22/094–3295) and complied with good clinical practice guidelines, the Declaration of Helsinki, and the applicable local laws.

## Consent

Given its retrospective design, the study was exempted from obtaining informed consent.

## Conflicts of Interest

The authors declare no conflicts of interest.

## Supporting information


**Figure S1:** Schematic treatment diagram of enrolled patients.

**Figure S2:** . The landmark analysis of progression&free survival (PFS) and overall survival (OS) of patients with advanced HCC, both PFS and OS measured from radiotherapy initiation. (a) Survival outcomes at 6 months. (b) Survival outcomes at 12 months. (c) Survival outcomes at 18 months. (d) Survival outcomes at 24 months. OS, overall survival; PFS, progression&free survival.

**Figure S3:** Survival analysis of patients with advanced HCC, both PFS and OS measured from regorafenib initiation. (A) PFS of patients with concurrent regorafenib treatment versus sequential regorafenib treatment. (B) OS of patients with concurrent regorafenib treatment versus sequential regorafenib treatment. PFS, progression‐free survival; OS, overall survival; HR, hazard ratio; CI, confidence interval; HCC, hepatocellular carcinoma.

**Figure S4:** Univariate analysis for PFS and OS of patients with advanced HCC, both PFS and OS measured from regorafenib initiation. PFS, progression‐free survival; OS, overall survival; HR, hazard ratio; CI, confidence interval; BCLC, Barcelona Clinic Liver Cancer; PVTT, portal vein tumor thrombus; ALBI, albumin‐bilirubin; HCC, hepatocellular carcinoma; AFP, alpha‐fetoprotein.

**Figure S5:** Liver MR scan of case 1. A male in his early 40s presented with gastric bleeding and a primary hepatic lesion located in the right hepatic lobe with right portal vein involvement. He subsequently received lenvatinib, but PVTT progression (yellow arrow) was detected 1 month later (a‐c). The primary lesion and PVTT were treated with RT (PTV: 50 Gy/2 Gy/25 f; GTV: 60 Gy/2.5 Gy/25 f) and concurrent regorafenib (120 mg per day). Four months later, the primary lesion (red circle) and PVTT (yellow arrow) had disappeared completely (d‐f). DWI, diffusion‐weighted imaging; MR, magnetic resonance; PVTT, portal vein tumor thrombus; RT, radiotherapy; PTV, planning tumor volume; GTV, gross tumor volume.

**Figure S6:** Liver MR scan of case 2. A man in his early 50s presented with a recurrent hepatic lesion located in the right hepatic lobe, and major portal vein trunk involvement was observed during his regular imaging follow‐up. Thus, he received sorafenib (400 mg bid) and 2 TACE procedures. However, growth of the hepatic lesion was detected (red circle) (a‐c). Thus, he underwent RT (PTV: 20 Gy/4G y/5 f; GTV: 25 Gy/5 Gy/5 f) and regorafenib (120 mg per day). Six months later, no viable primary lesion or PVTT was detected (d‐f). DWI, diffusion‐weighted imaging; MR, magnetic resonance; TACE, transarterial chemoembolization; RT, radiotherapy; PTV, planning tumor volume; GTV, gross tumor volume; PVTT, portal vein tumor thrombus.

**Table S1:** Outcomes of second‐line regimens after first‐line treatment failure for advanced HCC patients.

## Data Availability

The data that support the findings of this study are available from the corresponding author upon reasonable request.
