# Supplementary material for: Efficacy and Safety of Intensity‐Modulated Radiotherapy Combined With Regorafenib With or Without Immune Checkpoint Inhibitors as Second‐Line Treatment for Advanced Hepatocellular Carcinoma: A Real‐World Cohort Study From a Single Center
Source: Cancer Med. 2026 Apr 10;15(4):e71745. doi: 10.1002/cam4.71745 (PMC13069142; doi:10.1002/cam4.71745)
Supplement: Supplementary file 7 — Table S1: Outcomes of second‐line regimens after first‐line treatment failure for advanced HCC patients. [file CAM4-15-e71745-s004.docx]

Supplementary Table 1. Outcomes of second-line regimens after first-line treatment failure for advanced HCC patients

| Study | Previous regimens | Treatment regimens | ORR | PFS (months) | OS (months) |
| --- | --- | --- | --- | --- | --- |
| RESORCE trial ^[6]^ | sorafenib | regorafenib | 11% | 3.1 | 10.6 |
|  |  | placebo | 4% | 1.5 | 7.8 |
| REFINE trial ^[19]^ | sorafenib | regorafenib  (160mg) | NA | 3.9 | 15.8 |
|  |  | regorafenib  (120mg) |  |  | 13.3 |
|  |  | regorafenib  (80mg) |  |  | 11.1 |
| CELESTIAL trial ^[20]^ | at least one systemic treatment | cabozantinib | 4% | 5.2 | 10.2 |
|  |  | placebo | <1% | 1.9 | 8.0 |
| REACH trial ^[21]^ | sorafenib ± other systemic treatment | ramucirumab | 7% | 2.8 | 9.2 |
|  |  | placebo | <1% | 2.1 | 7.6 |
| Changhoon Yoo ^[23]^ | atezolizumab-bevacizumab | sorafenib, lenvatinib, and cabozantinib | 6.1% | 3.4 | 14.7 |
| Mara Persano ^[24]^ | atezolizumab plus bevacizumab or lenvatinib | TACE, sorafenib,  cabozantinib, immunotherapy, and other therapies | NA | NA | 18.6 |
| Young Eun Chon ^[25]^ | atezolizumab plus bevacizumab | sorafenib | 8.3% | 1.8 | 7.5 |
|  |  | lenvatinib | 5.6% | 3.5 | 10.3 |
| Choong-kun Lee ^[26]^ | atezolizumab plus bevacizumab | sorafenib | NA | 2.3 | 6.3 |
|  |  | lenvatinib | NA | 4.0 | 8.0 |
|  |  | tyrosine kinase inhibitor (TKI) plus immune checkpoint inhibitor (ICI) | NA | 5.4 | 12.6 |
|  |  | regorafenib | NA | 3.6 | 9.7 |
|  |  | cabozantinib | NA | 5.4 | 11.2 |
| Stephen L. Chan ^[27]^ | ICI-based treatment | cabozantinib | 6.4% | 4.1 | 9.9 |
| Anthony B. El-Khoueiry ^[28]^ | atezolizumab plus bevacizumab | regorafenib plus pembrolizumab | 5.9% | 2.8 | NA |
|  | durvalumab, nivolumab, ipilimumab, and pembrolizumab | regorafenib plus pembrolizumab | 11.1% | 4.2 | NA |

ORR, objective response rate; PFS, progression-free survival; OS, overall survival; TACE, transarterial chemoembolization; ICI, immune checkpoint inhibitors; NA, not available.
